# Day Care Laparoscopic Cholecystectomy: Next Standard of Care for Gall Stone Disease

**DOI:** 10.4021/gr374w

**Published:** 2011-11-20

**Authors:** Lileswar Kaman, Javid Iqbal, Ishwar Bukhal, Divya Dahiya, Rajinder Singh

**Affiliations:** a General Surgery, Post Graduate Institute of Medical Education and Research, Chandigarh, India; b General Anesthesia, Post Graduate Institute of Medical Education and Research, Chandigarh, India

**Keywords:** Laparoscopic cholecystectomy, Daycare surgery, Gallbladder, Postoperative nausea and vomiting

## Abstract

**Background:**

To access the feasibility, safety and success of day care laparoscopic cholecystectomy in a tertiary center in India.

**Methods:**

This is a retrospective analysis of prospectively collected data between 2004 and 2009 from a tertiary center in north India. All patients of symptomatic gallstone diseases having age less than 70 years, American Society of Anesthesiologists (ASA) grade I and grade II, living within 20 Kilometers of the hospital, availability of a responsible adult care taker at home, access to a telephone and a means of transportation to hospital if needed, underwent laparoscopic cholecystectomy under the care of the two participating surgeons, were considered for day care laparoscopic cholecystectomy. Clinical and operative data were recorded prospectively. All patients were discharged 6 to 8 hours after surgery with the advice to contact the surgical team over phone whenever necessary or on the day after discharge.

**Results:**

A total of 602 laparoscopic cholecystectomies were performed over a period of 6years, among them 309 (51.32%) were operated on day care basis. Nine patients in day care procedure group had conversion to open procedure (5 due to distorted anatomy of calot’s triangle, 2 due to common bile duct stones, 1 due to bile duct injury and 1 due to bleeding from cystic artery stump). One patient had myocardial infarction and 3 had nausea and vomiting which failed to resolve by intravenous ondensteron and all these (13) patients (4.20%) needed unplanned admission to the hospital. Two hundred and ninety-six patients (95.79%) were discharged on same day.

**Conclusions:**

In conclusion day care laparoscopic cholecystectomy is feasible, safe and equally effective in selected patients in Indian setup.

## Introduction

Laparoscopic cholecystectomy is the gold standard for symptomatic cholelithiasis [1, 2]. Many medical centers around the world have performed day care laparoscopic cholecystectomy (DCLC) in recent years [3]. The low rate of adverse events or complications during the intraoperative or immediate postoperative periods further justifies the rapid growth of this day care procedure [4-6]. The results of DCLC are promising in the developed nations [6-8], but performing laparoscopic cholecystectomy on an outpatient basis is not generally accepted in the developing nations, due to lack of equipped day care centers and transport problems. This audit study was done to analyze the feasibility, safety and success of DCLC in a tertiary referral health center in North India.

## Material and Methods

This is a retrospective analysis of prospectively collected data over a period of 6 years, between 2004 and 2009 from a tertiary health center in North India. DCLC was offered to all patients with symptomatic, ultrasound proved gallstone disease who met the following inclusion criteria’s: Patients willing for daycare surgery, age between 18 to 70 years, American Society of Anesthesiologists (ASA) grade I and grade II, BMI < 35 kg/m^2^, no history of jaundice, living within 20 Kilometers of the hospital, availability of a responsible adult care taker at home, access to a telephone and a means of transportation to hospital if needed.

The following patients were excluded: Patient having acute cholecystitis, ASA grade III or IV, outstation patients with no residence in vicinity of hospital, suspected or confirmed common bile duct calculi and patients on anticoagulant drugs.

All patients of gall stone disease presented at surgical outpatient department were evaluated by detailed history and complete physical examination. Those who had sign and symptoms suggestive of symptomatic gall stone were subjected to blood investigations like complete blood count, liver function tests, renal function tests and radiological workup including CXR and ultrasound abdomen. All patients were sent to anesthesia clinic for general anesthesia evaluation. After getting clearance from anesthesia clinic, patients were fixed for surgery. On the day before surgery, the patient with his/her family member returned to the outpatient clinic, where they were asked to sign a consent form for the surgery after discussion of the benefits and risks of the procedure. And instructions were given regarding surgery. All patients were advised to take tablet diazepam 10 mg and tablet ranitidine hydrochloride 150mg along with tablet metoclopramide 10 mg at 2200 hours night before the surgery and to repeat same drugs at 06.00 hours on the day of surgery as pre-medication. On the morning of the operation day, the patient arrived at the preoperative preparation room one hour before the planned operation after fasting for more than 6 hours. All day care patients were scheduled early in the morning list. All surgeries were performed under general anesthesia with endotracheal tube intubations.

General anesthesia was used with established following protocol. Fentanyl 2 mg/kg, Propofol 2 mg/kg and Vancuronium 0.1 mg/kg during induction and intubation and maintained by Oxygen and Nitrous oxide_._ Isoflurane, Vancuronium and Fentanyl boluses as and when required.

Trocar site were infiltrated with 2% lignocaine prior to incision. The surgery was performed by two senior consultant surgeons. Injection Cefazolin 1 gm slow IV was administered after test dose as prophylactic antibiotic 30 minutes prior to incision. Laparoscopic cholecystectomy was performed by standard American technique. Pneumoperitonium was created with the help of Veeres needle in 290 patients and in 19 patients with open technique due to umbilical hernia in 13 patients and lower midline surgical incision involving para-umblical region in 6 patients. CO2 was insufflated for distension of abdominal space followed by standard four port entry. Thereafter, Calots triangle dissected, cystic duct and artery clipped and divided, and gall bladder dissected off the gallbladder fossa by hook electrocautery in 271 patients and by using harmonic shear in 38 patients. Patients were shifted to postoperative recovery room and maintained on intravenous fluids for 4 hours post surgery. Patients were assessed at regular interval by a member of the surgical team and attending nurse for post operative complaints and vital signs. Injection Diclofenac sodium and injection Ondansteron were given for pain and nausea or vomiting if required. After 4 hours, operating surgeon along with anesthesiologist evaluated the patient for pain, nausea, vomiting, consciousness level and vital parameters (including oxygen saturation). They were encouraged to sit up, drink and go to the toilet under supervision. Four factors including nausea, vomiting, incisional pain and shoulder tip pain were critically evaluated.

Patients were discharged 6 to 8 hours after surgery if they satisfied the following criteria: 1) Vital signs were within 20% of preoperative level; 2) Patients were able to understand instructions and can ambulate; 3) Patients were relieved of nausea, vomiting and pain; 4) Able to tolerate liquids and void urine; 5)No bleeding from surgical sites; 6) Patient feeling comfortable and ready to go home willingly.

Patients undergoing daycare laparoscopic cholecystectomy were admitted if: 1) There was a conversion to open cholecystectomy; 2) If discharge criteria were not satisfied; 3) Unexpected medical problem attributed to the surgery.

Patients were given tablet Diclofenac sodium 50 mg orally three times daily and tablet Ondensetron 4 mg orally two times. Patients were provided phone number of the resident in charge and advised to contact if required or to report to the 24 hours emergency services if necessary. They were called back the next morning to assess general well being, pain, discomfort, nausea, vomiting or any other side effects attributable to anesthesia or surgery.

## Results

Over a period of 6 years, two participating surgeons did a total of 602 laparoscopic cholecystectomy for symptomatic gall stone disease. Out of these patients 309 fulfilled the inclusion criteria and underwent laparoscopic cholecystectomy on day care basis. Indications for surgery in these patients were recurrent biliary colic in 217 (70%) and previous episodes of acute cholecystitis in 92 (30%). Patient’s profiles are shown in Table 1. Total of 293 patients were excluded from day care laparoscopic cholecystectomy group, reasons for exclusion are shown in the Table 2. Unplanned admissions were required for13 patients, among them conversion to open cholecystectomy in 9 patients, obscured calot’s triangle anatomy in 5 patients, bile duct injury in 1 patient, common bile duct injury in 2 patients and bleeding from cystic artery in 1 patient. One patient had myocardial infarction and 3 had nausea and vomiting which failed to resolve by intravenous ondensteron and all these (13) patients (4.20%) needed unplanned admission to the hospital. Two hundred ninety-six patients were discharged on the same day within 6 to 8 hours of surgery. Assessment of postoperative pain, nausea and vomiting shown in Table 3.

**Table 1:** Patients Characteristics in DCLC Group (n = 309)

S. No.	Patients characteristics	Value
1	ASA grade 1	230 patients
2	ASA grade 2	79 patients
3	Age (Range)	18 - 70 years
4	Sex (F:M)	270:39

**Table 2:** Patients Excluded From DCLC Group (n = 293)

S. No.	Causes of exclusion	Patients number
1	Patient resident out of stipulated area	219
2	ASA grade 3	18
3	Age > 70 years	16
4	Patient not willing	17
5	Poor social support	10
6	Acute cholecystitis	13

**Table 3:** Assessment of Postoperative Pain, Nausea and Vomiting

	Time of discharge	Next day
Incisional pain		
Mild	61 (19.8%)	49 (15.86%)
Moderate	240 (77.6%)	260 (84.14% )
Severe	8 (2.6%)	0
Shoulder tip pain		
Nil	154 (49.8%)	230 (74.43%)
Mild	140 (45.3%)	78 (25.24%)
Moderate	15 (4.9%)	1 (0.4%)
Severe	0	0
Nausea		
Mild	41 (13.85%)	4 (1.2%)
Moderate	78 (26.54%)	2 (0.6%)
Severe	4 (1.2%)	0
Vomiting		
Mild	34 (11.48%)	3 (0.97%)
Moderate	17 (5.74%)	3 (0.9%)
Severe	1 (0.4%)	0

Mild: No medication; Moderate: Oral medication; Severe: IM/IV medication.

Three patients required readmission in the postoperative period, 2 for intraabdominal collection and 1 for fever. Pigtail catheter drain done in both the patients for intraabdominal collection, no patient required surgical intervention. There was no mortality during perioperative and follow up period.

## Discussion

Daycare laparoscopic cholecystectomy (DCLC) has recently been adapted as a safe and viable procedure and is rapidly gaining popularity because of cost saving and convenience. The low rate of adverse events or complications during the intraoperative or immediate postoperative periods further justifies the rapid growth of this type of ambulatory surgery in developed nations [5, 6, 9, 10]. All these data are coming from advanced countries where already there is a system for ambulatory or day care surgery is in place. Also well defined inclusion and exclusion criteria are followed for patient selection. But data from developing nations like India is still limited. The experience from India has reported it to be safe, feasible, and acceptable to patients and with social and economic benefits [10-15]. Performing DCLC in high risk patients presents a challenge to surgical safe practice, particularly during the early postoperative period. Saunders et al [16] has reported mortality after DCLC there by advocating caution before performing this procedure in day care setting. Performance of DCLC in high risk patients requires scrupulous evaluation prior to implementation [17, 18]. Criteria for patient’s selection are crucial for the development of safe day care surgery. Robinsons et al [19] reported to have achieved success of 70% of an unselected group of patients and they have identified ASA classification, procedural duration and surgery start time as factor associated with failure of outpatient management. It has been concluded in studies that appropriate patients selection lowers failure rate and patients most likely to fulfill the criteria of DCLC are patients of ASA grade I and II, with no previous abdominal surgery, no history of acute cholecystitis and a procedural duration of shorter than 90 min [7, 12, 20]. Most studies utilize selection criteria when evaluating patients for DCLC [5, 21]. Ali et al [22] reported successful DCLC in 92% of selected patients. In our study only patients who fulfill our selection criteria were subjected to DCLC and resulted in successful completion of DCLC in 96% patients. The rate of unplanned admission in DCLC is a quality index as it might represent the existence of inadequate criteria in selection of patients who given their characteristics, precedents, or preoperative findings were not candidate to this type of surgery. A lower admission rate has been reported in freestanding ambulatory surgery centers and this could be related to their strict patient's selection criteria [20-22]. Our unplanned admission rate of 4.2% in present study compares favorably with the results of other centers in appropriately selected patients. Most important cause for failure to discharge in our study was conversion to open surgery followed by refractory nausea and vomiting. Hollington et al [23] reported postoperative nausea and vomiting a frequent reason for unplanned admission after DCLC. The frequency of post operative pain, nausea and vomiting in our study was remarkably less, which might have occurred due to proper patient selection and use of adequate preoperative analgesia, anxiolytics and prokinetic agents. In our study only three patients required readmission in the postoperative period and no patient was reoperated and there was no mortality. The limitations of the study are, it is a retrospective report with prospectively kept data and no analysis of the patient satisfaction was done. May be this aspect may be studied in future studies.

### Conclusion

Day care laparoscopic cholecystectomy is feasible, safe and equally effective in India if performed in selected group of patients after establishing strict patient selection criteria.
